# Australian Stakeholder Perspectives on Healthier Retail Food Environments for Toddlers—The Era of “Band Aids and Small Inroads”

**DOI:** 10.1016/j.cdnut.2023.102060

**Published:** 2023-12-09

**Authors:** Jennifer R McCann, Julie Woods, Catherine G Russell

**Affiliations:** Deakin University, Geelong, Australia, Institute for Physical Activity and Nutrition (IPAN), School of Exercise and Nutrition Sciences

**Keywords:** health promotion, policy, public health, qualitative, regulation, retail food environment, stakeholder, toddlers

## Abstract

**Background:**

A healthy diet in young children is crucial for optimal growth and development. However, many toddlers (1-3 y) consume suboptimal diets, and as a result, are at a high risk of experiencing negative health outcomes. Moreover, minimal progress has been made to improve the healthiness of retail food environments for toddlers to date despite the potential and advocacy for the issue.

**Objectives:**

To gain insight into stakeholder perceptions and opinions on the healthiness of Australian retail food environments for toddlers, as well as perspectives on the options and barriers to improve their healthiness.

**Methods:**

Qualitative, online study utilizing semi-structured individual interviews with 27 key stakeholders from food industry, academia, nongovernment organizations, public health, and government in Australia.

**Results:**

Most stakeholders agreed that retail food environments for toddlers were not health promoting. Stakeholders identified that a multifaceted approach including nutrition education and strong government mandated regulation were essential to improve the healthiness of retail food environments for toddlers. Interviews also highlighted the main perceived barriers to progress, and reasons for policy inaction in this area are the food industry and government support. Many stakeholders were concerned that child health is being undermined due to the government favoring business needs over public health.

**Conclusions:**

Stakeholders in this study overwhelmingly agreed that there is more that can and should be done to create health promoting retail food environments for toddlers in Australia. Stakeholders identified a range of strategies that can be used to improve the healthiness of toddler food environments, but advocacy efforts are being undermined due to government inaction. Stakeholders believed that strong governance is required to create equitable, sustainable healthy retail food environments for young children. Improving the healthiness of retail food environments for young children will not only reduce diet related disease across the lifespan but will help to address financial and societal costs of a poor diet.

## Introduction

A healthy diet in early childhood is essential to provide the foundation for optimum growth and development [1]. While family foods progressively replace breastfeeding and/or infant formula across the second 6 mo of life, WHO recommends exclusive breastfeeding until 6 mo of age and continued breastfeeding until 2 y of age and beyond, alongside healthy family foods [2]. Despite this, suboptimal diets high in ultra-processed (UP) foods [3] and low in vegetables [4] are prevalent in toddlers (aged, 1-3 y). UP foods are defined as “formulations of ingredients, mostly of exclusive industrial use, that result from a series of industrial processes” [5]. This has resulted in an increased risk of negative health outcomes related to dietary intake [6]. A contributing factor to the poor diet quality seen in toddlers is the steady increase in the global sales and availability of UP foods and drinks specifically for the toddler age group [7,8].

Efforts to promote healthy dietary behaviors in early childhood are being undermined by non-health promoting retail food environments, which include stores such as supermarkets, chemists, or where toddler food and milk is sold in a retail capacity and associated advertising and promotional activities. This is highlighted by a recent Australian audit which reported that 85% of toddler specific foods, and 100% of toddler milks being sold in retail stores are UP [7].

In addition, there has been a noticeable increase in UP foods and milks for toddlers on Australian supermarket shelves [9]. Research has reported that globally, UP foods are also a significant source of dietary energy for toddlers [10]. In Australia there is a concentration of manufacturer and retailer power [11]. This concentration of power largely determines packaged food availability, and as a result, restricts food choices for consumers, while simultaneously providing an illusion of choice. This highlights the crucial role food manufacturers and retailers have in improving retail food environments and preventing diet related disease.

The WHO supports policy action to improve the healthiness of retail food environments for young children (which includes infants 6 to 12-mo-old and toddlers 12 to 36-mo-old) [12]. The WHO is calling upon food and advertising agencies to end inappropriate forms of promotion. In the context of the current study, this includes: a) the promotion of products high in sugar, salt and fat (which contribute to childhood obesity and non-communicable disease); b) foods not recommended within national dietary guidelines (often UP); and c) misleading on-pack labeling (product name, use of nutrition claims) [13]. The WHO is also encouraging all countries to “promote policy, social and economic environments that enable parents and caregivers to make well informed infant and young child [toddler] feeding decisions, and further support appropriate feeding practices by improving health and nutrition literacy” (P.34) [12]. It is essential to identify potential policy opportunities and pathways to inform positive and meaningful change to retail food environments for young children to improve nutrition and diets in the toddler years.

Currently, there is no specific regulation in Australia for toddler foods, other than regulations that apply to all foods [14], while infant foods [15] and formulas [16] have their own specific regulations (FSANZ standards 2.9.2 and 2.9.1 respectively). Toddler milks are able to be sold by being considered a formulated dietary supplement for young children with special dietary needs within Food Standard Australia New Zealand standard 2.9.3 [17] although they are advertised for use by healthy toddlers. The current permissive food policy and regulation in Australia is enabling marketing loopholes, and potentially misleading consumers.

Despite significant research underpinning the need for investment and action into retail food environments for toddlers, fragmentation among stakeholders is a persistent problem, resulting in policy inaction [18]. Stakeholders often hold contrasting opinions on preferred actions to improve diets in the early years due to their vested interests as well as personal experiences and values. In Australia there has been little progress over the last decade toward policies that support healthy retail food environments for toddlers [19]. With no clear targets, competing stakeholder views, in addition to internal impediments to progress (i.e. agreement about what constitutes an unhealthy food), political action in this area has been slow [20]. In Australia, the creation of policy and regulation relating to toddler retail food environments is influenced by stakeholder perspectives and opinions from public health and nutrition organizations, relevant non-government organizations (NGO), academics, federal and state government, members of Food Standards Australia New Zealand, and the food industry (FI), including retailers.

This study aimed to examine stakeholder perceptions and opinions of the healthiness of retail food environments for toddlers in Australia, options to improve their healthiness, as well as potential barriers that would hinder progress or action.

## Methods

Semi-structured individual interviews were conducted with stakeholders. The study was conducted and reported according to the Consolidated Criteria for Reporting Qualitative Research [21]. Ethics approval was provided by the Deakin University Human Ethics Advisory Group (HEAG-H 164_2021). The interviewer (JM) held a relativist ontological position using an epistemology which embraced subjectivity, and gave equal value to all stakeholder responses. For the purpose of this study, retail food environment was defined as the retail spaces where food is sold (e.g., supermarket, pharmacy) and associated with advertising and promotional activities, a toddler includes children aged 1 to 3 y), and a retail food environment for toddlers includes both toddler foods and milks.

### Sample and recruitment

Stakeholders were identified through personal networks and internet searching and were defined as individuals working within a relevant area, such as the FI, public health, food policy, or early childhood, (minimum 5 y), or someone with significant knowledge and/or expertise relative to early childhood or toddler retail food environments. Food Industry representatives were specifically included in this study, as they have detailed insight into what is possible from a product perspective, as well as potentially having a very polarizing viewpoint from other stakeholders, which was important to capture in this research.

To ensure sample diversity and include a range of opinions and perspectives, this included members of the FI (specifically only nutritionists or dietitians working for a company, including retailers, which manufactured or sold toddler foods or milks), academia, NGOs, public health, and nutrition groups, as well as state and national government. Purposive and snowball sampling was undertaken to contact participants. All communication was via email. Stakeholders were emailed with a study overview, the Plain Language Statement and details on how to participate. Interviews were then scheduled if they agreed to participate, which was indicated via return email. If they declined, no further contact was made. JM followed up if no reply was received within 2 wk, after which no further contact was made. A similar number of stakeholders were to be contacted within each group, but due to the small number of NGOs relevant to the topic, fewer from this group were contacted. Owing to the small sample of experts in this area, there were some unavoidable pre-existing relationships between the interviewee and stakeholders. It was made very clear in the study information sent to stakeholders before the interview that participation was voluntary. In addition, many stakeholders sent the study invite to colleagues or others they felt would be more suitable if they were unable or did not wish to participate, thereby eliminating the potential for any conflicts of interest. See Table 1 for an overview of key stakeholder participation.

**TABLE 1:** Description of stakeholders recruited for interviews.

Stakeholder group	Number of stakeholders				
	Contacted	Replied	Declined	Accepted	Interviews conducted
Food industry (including retail)	18	11	2	9	8
Government	18	10	6	4	4
Nongovernment organizations	6	6	1	5	4
Academia	15	9	4	5	4
Public health/other nutrition	17	11	3	8	7
Total	74	47	16	31	27

### Data collection

Individual semi-structured interviews, which included prompts based on individual responses to enabled authentic interviews to be conducted based on each stakeholder’s experiences, views and perspectives. All interviews were conducted online (video and audio recorded) by a trained qualitative researcher (JM) through Microsoft Teams, between May and July 2022. The interview guide included predefined questions and probes to prompt participants to elaborate on their responses, which allowed for a conversational style interview to occur. All participants provided verbal consent to participate at the commencement of the interview. Transcripts were not provided to participants for comment.

### Semi-structured interview guide

The interview guide was developed with input from all authors and was pilot tested with a small convenience sample. As there has been minimal progress and research on toddler retail food environments, as well as few key stakeholders working exclusively with/on the toddler age group, some questions were asked more generally about young children (which refers to ages 6 mo to 5 y), with the notion that ideas and suggestions could be adapted to the toddler age group. The 3 main questions are shown below, and the complete interview schedule is in Supplemental File 1.

Q1 Do you feel that the current status of policy and regulation of toddler foods and milks adequately promote healthy retail food environments for young children in Australia?
Q2 What do you think are some policy options to promote healthier retail food environments for young children?
Q3 Do you think there would be any barriers to the options you have mentioned? Do you think there are any stakeholders or groups that may oppose the options you have suggested?

### Data analysis

All interviews were auto transcribed in Teams. JM checked the accuracy of the transcripts against the recordings, which were then deleted, and interview transcripts de-identified. The interviewer (JM) took detailed notes throughout each stakeholder interview, noting any key points or themes which emerged at the end of each interview. Interview transcripts were uploaded to NVivo (QSR International version 1.6.1) qualitative research software. Thematic analysis outlined by Braun & Clark [22] was performed by JM, using an inductive reasoning approach [23,24]. The first step according to Braun & Clark involved repeated reading of each interview transcript and associated notes, thereby familiarizing oneself with the data. Step 2 involved inductively coding the data (creating codes from the data). Step 3 consisted of identifying themes and subthemes. These themes and subthemes were then reviewed in Step 4. Step 5 involved clearly naming and defining each theme and sub-theme, and the final step involved writing up the results. Concise summaries of key themes were presented as key stakeholder quotes, which were de-identified, and only described according to their stakeholder group (i.e., Food Industry/Public Health etc.) in order to ensure participant confidentiality. To explore different interpretations of the data, during Step 2 [25], a random subset (10%) of transcripts was independently coded by the co-authors (JW and CR). All authors then met to discuss any discrepancies and final themes and subthemes were agreed upon.

The Health Impact Pyramid [26] and the Ecological model for barriers and opportunities for healthy eating [27], were adapted and used to categorize stakeholder responses to Questions 2 and 3 respectively in the deductive phase of analysis as they aligned with the responses from stakeholders as well as provided a broader perspective of the options and barriers stakeholders discussed.

## Results

The final sample included 27 individual stakeholders (Table 1). Only one stakeholder was male, and the mean interview duration was 45 min.

Q1 Do you feel that the current status of policy and regulation of toddler foods and milks adequately promote healthy retail food environments for young children in Australia?

With the exception of a few FI stakeholders who felt that toddler retail food environments were adequately regulated and health-promoting, the majority of stakeholders used terms such as “inept,” “insufficient,” “compromised by industry,” “profit over health,” and “unhealthy” when describing toddler retail food environments in Australia. “*In terms of the current landscape, to me it’s a confectionary aisle, it’s a mess, and I feel sorry for parents who are picking these products” (FI stakeholder).* A public health stakeholder highlighted the specific lack of regulation for toddlers “*It's definitely lacking in terms of specific regulation for children aged 12-36 months.”*

Q2 What do you think are some policy options to promote healthier retail food environments for young children?

**FIGURE 1:**
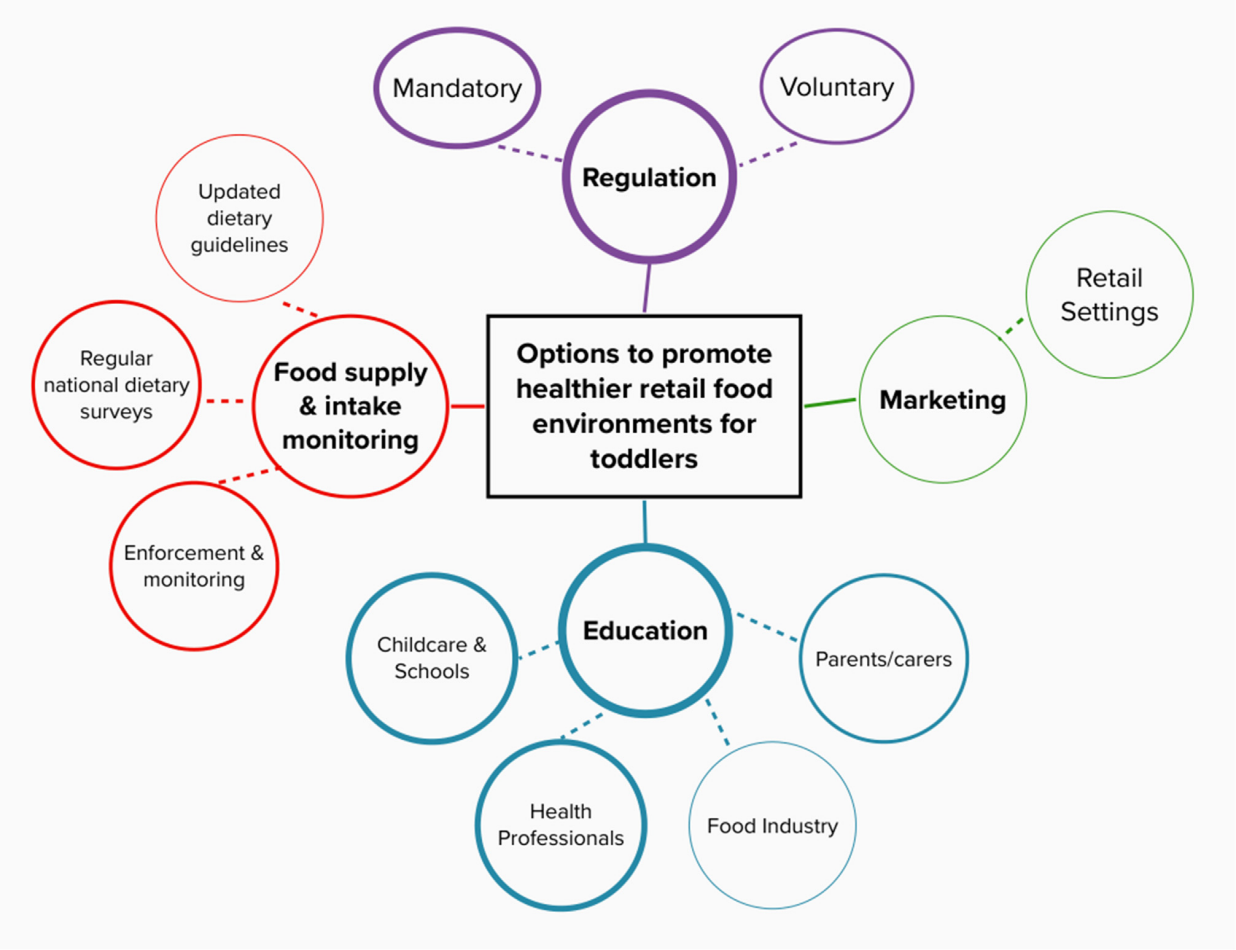
Policy options suggested by stakeholders to promote healthier retail food environments for toddlers in Australia. Each color indicates a different theme. Thickness of lines correlates to more stakeholders mentioning this option.

All stakeholders discussed options that could be implemented to create healthier retail food environments for toddlers. See Figure 1 for a visual representation of the options suggested.

## Regulation

### Mandatory regulation

Stakeholders mentioned mandatory regulation, which included plain packaging, restricting health and nutrition claims, removing on-pack cartoons, and implementing warning labels for nutrients of concern (sugar and sodium) and level of processing. Many stakeholders referred to the current situation of on-pack marketing and regulation of toddler foods and milks as misleading, unethical, deceptive, and problematic. Introducing compositional limits for sugar and sodium was mentioned by most FI stakeholders. “*Mandatory regulation is needed to adequately protect children. I think there are lots of other policy options that would slightly improve the situation, but they're all kind of like little band aids and small inroads. I think to effectively create a healthy [retail] food environment for young children in this packaged food space, there needs to be mandatory regulation” (NGO stakeholder).* Some FI stakeholders supported mandatory regulation, discussing how regulatory compliance is an issue with voluntary regulation “*Voluntary targets can have issues with compliance. If companies choose not to adhere, it creates an unfair playing field.”*

### Voluntary Regulation

Voluntary regulation was a common theme among FI stakeholders, with The Healthy Food Partnership early years subgroup (a working group of government, public health and the FI who are working toward finding solutions to enable consumers make healthier food choices) mentioned as a key government action, and voluntary industry guidelines as an important outcome. “*I do feel that there is a space for some industry voluntary guidelines, which is what The Healthy Food Partnership is trying to develop” (FI stakeholder).*

Although already being permitted, implementing the Health Star Rating on toddler foods, and encouraging reformulation to gain higher ratings was mentioned as well as fortification of toddler foods with iron to address potential nutrient deficiencies in this age group.

### Nutrition Literacy Education

Many stakeholders stated that nutrition education would not be an effective standalone strategy, but would play a role in a multifaceted approach to any policy relating to creating healthier food environments. While education may not be specific to retail food environments, stakeholder responses made it clear how they are just one component of an effective policy option. “*Education's always an important component, you can educate till you’re black and blue in the face. But if you send someone out into an environment where these products are readily available, the healthy choice is not the easy choice. The easy choice is what's the shelves are laden with. Education, absolutely. However, not education alone. It does not equate to a significant change in behavior when the environment* *supports* *otherwise” (government stakeholder).*

Stakeholders discussed the need for practical, tailored education for parents and carers, including education around provision of appropriate foods for toddlers, as well as education around fussy eating. “*We need education for parents around healthy eating messages and also how to make it work within busy lifestyles” (government stakeholder).* Several stakeholders also discussed educating health professionals, including General Practitioners, pediatricians, dietitians, and maternal and child health nurses around healthy eating for toddlers, as they are often seen as reliable sources of information in addition to having a wide reach. *“Professional education for pediatricians, maternal, and child health nurses and even dietitians around the appropriate use of these products. They are that first port of call and have so much power to change and influence the way people are feeding their children” (FI stakeholder).* Stakeholders also discussed nutrition education within schools and childcare centres, with suggestions such as cooking classes, menu planning resources, food provision policies, and even additional home economics classes. “*I think the toddler food-industry represents the systemic issue of our society; therefore, education needs to be in all schools so that when these students become parents, they have the skills to feed their families” (public health stakeholder).*

### Stricter marketing rules

Cited by all stakeholder groups except NGO, was stricter rules around on-pack and mainstream marketing of toddler foods and milks. Stakeholders discussed the lack of perceived government support and subsequent political will for food marketing restrictions, with one academic stakeholder noting strong action to restrict unhealthy food marketing to young children as a critical step for progress. “*We need political will. We need a government led, mandated, comprehensive approach to restricting all unhealthy food marketing that children are exposed to, or for foods that are intended for consumption for children.”* Retail settings in particular were mentioned as an area for policy action, “*retail settings are where you make your purchasing decisions. And whilst it looks good that Woolies and Coles have their free fruit for kids, when you go into the supermarket, at the end of every single aisle, it's just full of lollies and chocolates and soft drinks…supermarkets, or retail settings are an area where parents are getting information” (government stakeholder).*

### Food supply and intake monitoring and enforcement

Regular monitoring of the food supply and population dietary intakes was mentioned by participants from all stakeholder groups, but by more FI stakeholders than others. Some discussed at length the concern that our food supply has expanded, and the current food standards codes have not been able to keep up. “*When were these regulations written and what did the food space look like then? Because maybe they are just really out of date, or when they were written, they were actually not so bad, but now because the [retail] food environment has changed so much, the regulations haven't kept up” (public health stakeholder).* Regular national health surveys which would result in more accurate and up to date data on toddler diets was also discussed by stakeholders. In addition, more frequent updates to the Australian Dietary Guidelines, including the infant feeding guidelines were mentioned, as these are often the key sources of evidence Food Standards Australia New Zealand (FSANZ) uses. *“It's now 10 y, and a lot of research has changed and that has a knock-on effect for FSANZ, who still use the science reflected in those guidelines” (FI stakeholder).* Except for the FI, many stakeholders discussed enforcement and meaningful implications for breaches to the food standards code to enhance the current regulatory status quo. “*The other powerful lever for changing systems is creating feedback mechanisms, and this is where strong monitoring and enforcement systems which are well-funded, and highly resourced with experts are needed” (academic stakeholder).*

**FIGURE 2:**
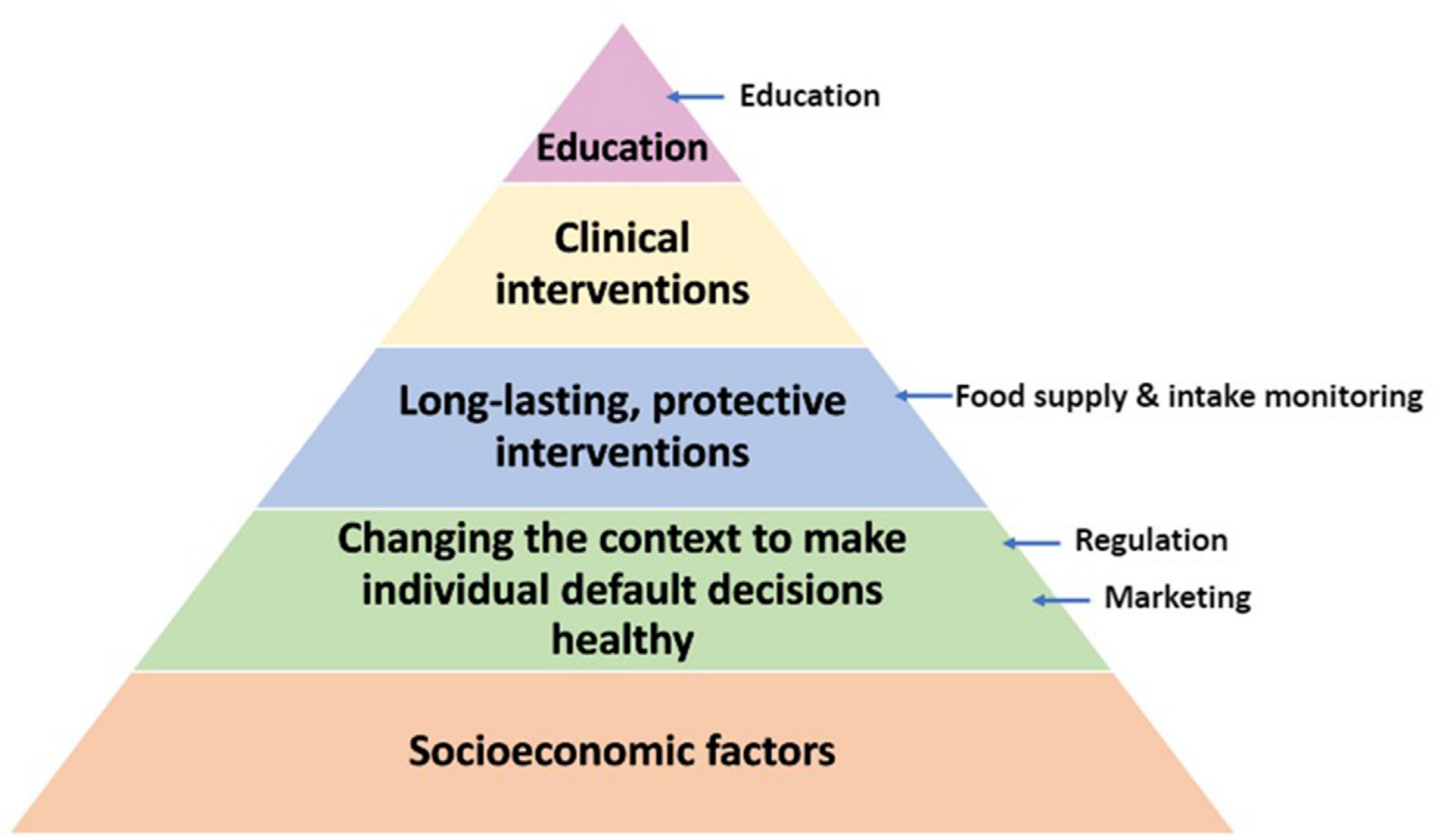
Health Impact Pyramid [26] adapted to include stakeholder options to improve the healthiness of toddler retail food environments in Australia.

Utilizing the Health Impact Pyramid [26] (Figure 2) to categorize key stakeholder responses from Question 2, it can be seen that no options suggested by stakeholders address socioeconomic factors, with education having the least population health impact and requiring a high level of individual effort.

Q3 Do you think there would be any barriers to the options you have mentioned? Do you think there are any stakeholders or groups that may oppose the options you have suggested?

**FIGURE 3:**
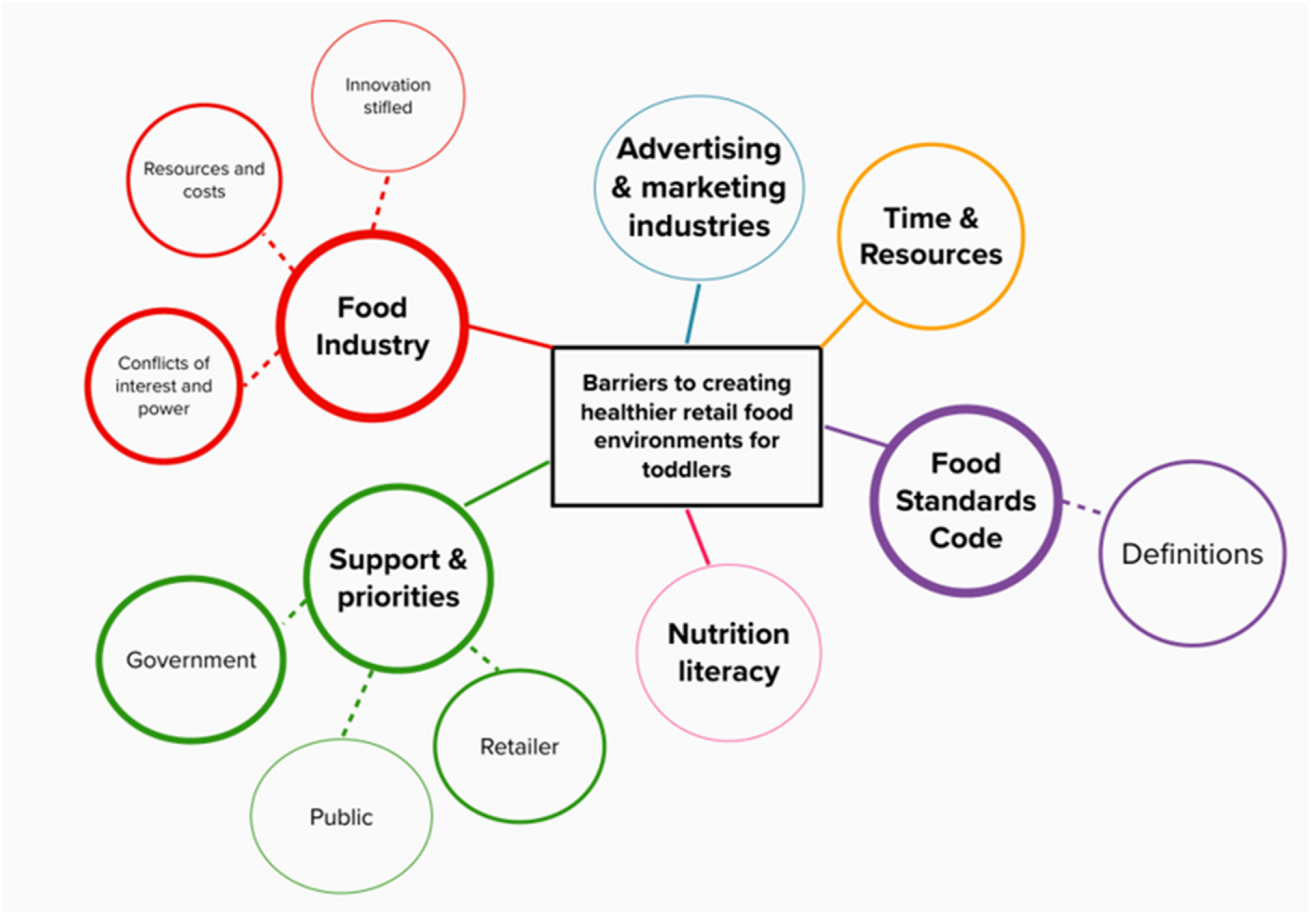
Barriers to creating healthier retail food environments for toddlers as mentioned by stakeholders. Each color indicates a different theme. Thickness of lines correlates to more stakeholders mentioning this barrier.

Potential barriers or opposing stakeholder groups mentioned by stakeholders were wide ranging. See Figure 3 for a visual representation of these.

### Food Industry

The FI was mentioned by all stakeholder groups(including the FI stakeholders), as a barrier to creating healthy retail food environments for toddlers. For reference, the FI often includes food manufacturers, retailers and associated marketing and advertising industries, however, marketing and advertising industries were considered a separate entity for this study as they have their own codes of conduct around advertising to children. There was some tension between stakeholder responses from FI compared to other groups, as many non-FI stakeholders discussed the conflicts of interest within government led “public health” groups (e.g., The Healthy Food Partnership), as well as the clear imbalance of power and resources between the FI and public health. “*Food-industry has far too much power and influence on any attempt to implement regulation. They often are very experienced, powerful and wealthy lobbyists. And we have such a tiny public health workforce. We are a drop in the ocean compared to everything that they can throw at us. So a major barrier is the wealth and influence and experience of industry and their ability to lobby” (government stakeholder).* Many stakeholders, including some from FI, felt that while the FI should be *involved* in initial discussions, they spoke at length that the FI should not be actively contributing to policy decisions around early childhood feeding, with one academic stakeholder having this to say “*We can follow what has been done in tobacco control. To firewall industry from participating in infant and toddler feeding policy decision making…Representatives from the food-industry should recuse themselves, and not be involved in decision making.”* Meanwhile, most FI stakeholders mentioned barriers such as costs and resourcing if there were any requirements for product redevelopment or label compliance. Some FI stakeholders also discussed the possibility of innovation being stifled if too much regulation was imposed, “*One argument about prescribing mandatory composition limits is that it can stifle innovation, and so if you have very strict laws on toddler foods, companies are sometimes thwarted as to their innovation.”*

### Advertising and marketing industries

Some stakeholders mentioned the advertising and marketing industries as a barrier to progress, as they often are highly resourced and skilled in lobbying against anything which would result in financial implications or potential loss of revenue, as these industries operate on a profit basis. “*So we know that the advertising industry will push back. Any industry with a conflict of interest who are making money out of this kind of marketing.” (academic stakeholder).*

### Insufficient Support and Priorities

Insufficient support from government, the public, and retailers was a highly cited barrier across all stakeholder groups. Stakeholders used phrases such as “holding the purse strings,” and “standing up to food-industry” when discussing how lack of government support was a barrier. “*At the core of things, it is government, we've got multiple organizations in Australia advocating government for change in these spaces, and it's not happening. They're a really big barrier to change. They need to prioritize health” (NGO stakeholder).* Public support was also cited as a barrier and many stakeholders referred to brand loyalty in this aspect. “*The problem is that companies diversify their product range in such a way that they're marketed to families so that they might continue to purchase these products because they're marketed specifically for growing children” (public health stakeholder).* Stakeholders also discussed how lack of retailer support can be a barrier, with many perceiving retailers to be averse to risk when taking on new products or pack formats, as well as dictating the layout of retail space and specials and pricing, thereby influencing product availability and affordability and consumer awareness. *“Looking at for instance, those squeeze pouch tops. I've been wanting to move away from them for years, but that's what the retailer wants. It's the only format that they're accepting” (FI stakeholder)*.

A small number of stakeholders discussed the barrier of having too many public health issues ripe for action, which can stagnate progress on determining which are prioritized. *“There are a lot of issues with these products, and it’s sort of like how do you pick out the main ones to communicate as advocacy? Is it that there is too much sugar, they are ultra-processed, or are we worried that they're not having fruit and vegetables? There are so many different problems” (NGO stakeholder).*

### Food Standards Code

The majority of non-FI stakeholders mentioned the Food Standards Code as a barrier, as the relevant standards are either outdated or non-existent, in addition to any change to the standards taking a long time. “*When the Food Standards code was created there was no such thing as toddler foods, they didn't exist. And so it's about the standards keeping up with the pace of the market and that hasn't happened” (government stakeholder).* Stakeholders cited the need for a clear definition of added sugar, which is currently in progress in Australia and New Zealand, as this would impact progress on both voluntary and mandatory regulation. Stakeholders also cited uncertainty as to what foods could be defined as a toddler food, which would have implications for any new standard or policy action, as well as the need for consistent definitions around a definition of a healthy food, as this would have repercussions on how potential interventions are designed and policy action is prioritized.

### Nutrition literacy

Poor nutrition literacy among the general public was mentioned as a barrier across all stakeholder groups (except public health). Stakeholders felt that the public health tools, which are often used to convey healthy eating messages are not able to be personalized and are therefore not easily used by the general public, “*The dietary guidelines mean nothing to people who are not in the nutrition space. It's not going to make them eat something they don't have.” (academic stakeholder).* In addition, stakeholders also discussed the barrier of nutrition literacy in the context that most consumers are unable to correctly interpret nutrition information panels or product labels. “*Health literacy in general can be a barrier to people, not even understanding that they need to look at the nutritional panel, and the extra education that's needed if the label is changed and better, you still got to decipher it” (NGO stakeholder).*

### Resourcing and Time

A common barrier mentioned across stakeholder groups was resourcing, such as staffing and money, as many of the options stakeholders suggested were resource intensive. One government stakeholder discussed resourcing as a barrier to education within childcare settings *“I think that becomes a* *funding* *issue and a skills-based issue. Having people that have the skills to be able to prepare and to cook the food as well as making sure that the childcare costs are not increased to a point that is prohibitive.”* Time was mentioned as a barrier across all stakeholder groups, and included time for educational campaigns to run, for consumers to read and interpret on-pack labels, as well as time to get a new policy or regulation implemented. Some stakeholders discussed that any new standard developed would take so long that it may be outdated and not reflective of the current retail food environment. “*I think time frame would be one. I mean any kind of policy or regulatory change in particular is such a slow burn. The main group that would miss out is anyone who is going to be zero to three in the next 10 years, because it's going to realistically take more than that to get this over the line” (NGO stakeholder).*

**FIGURE 4:**
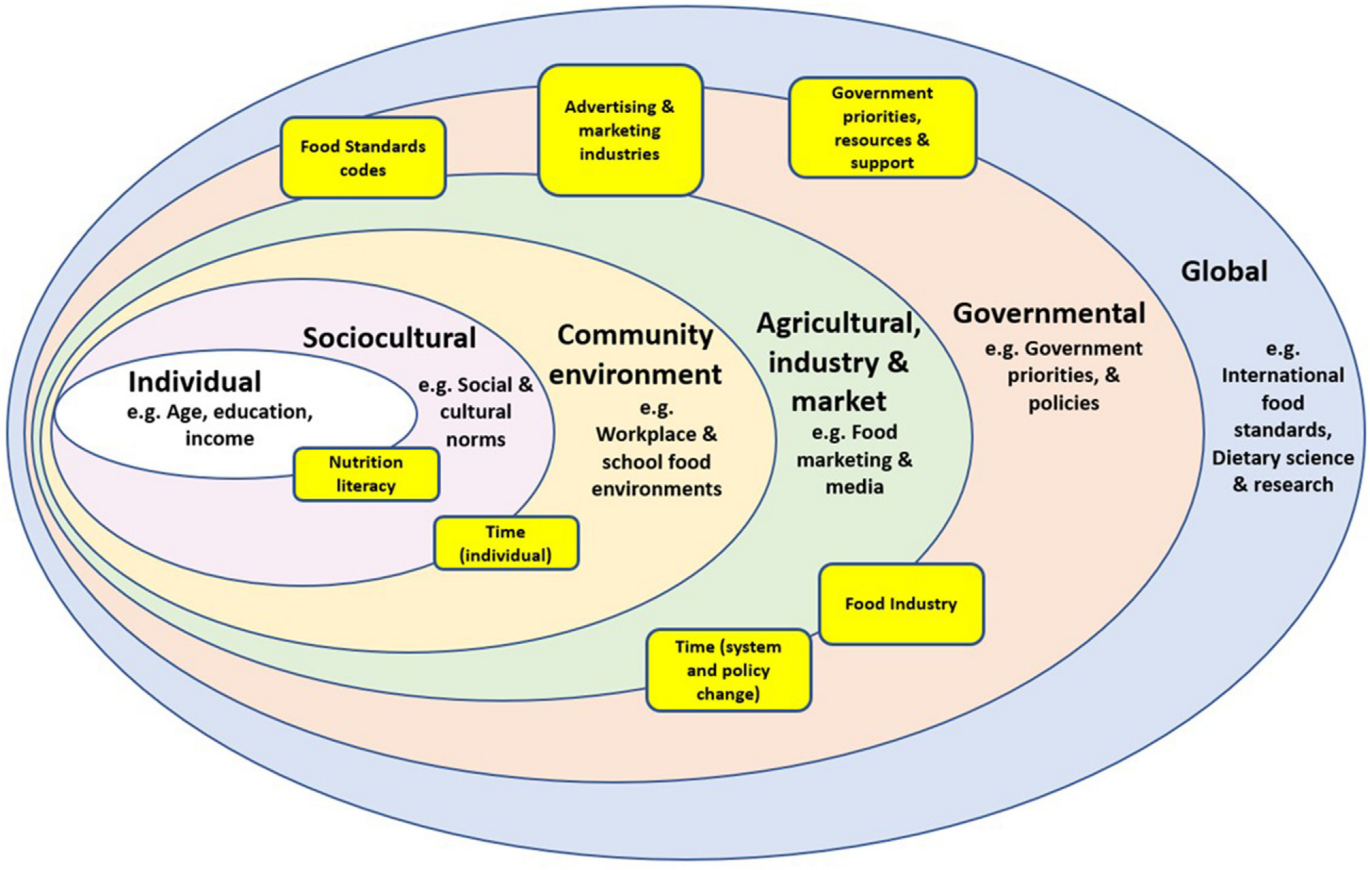
Ecological model of barriers for healthy eating for toddlers, adapted from Afshin et al. [27] (2014), demonstrating the dominant role of government in stakeholder perspectives. Rectangles represent barriers which stakeholders identified that may impede progress to improving the healthiness of toddler retail food environments in Australia. Rectangles that cover more than one domain indicate that they impact across multiple domains.

Referring to the Ecological model for barriers and opportunities for healthy eating [27], (Figure 4) the majority of the barriers mentioned by stakeholders would fit within the governmental or agricultural, industry and marketing domains, demonstrating how heavily food policy and regulation and governmental priorities influence food choice for toddlers, as well as how important a multifactorial strategy is to achieve an effective solution.

## Discussion

This study provides an in depth understanding of the opinions of key stakeholders regarding policy options and barriers to creating healthier retail food environments for toddlers in Australia. Stakeholders believed that a multifaceted approach including nutrition education as well as strong government regulatory action should be the priority. The current findings also highlighted that stakeholders believe the main barriers to progress in improving the healthiness of retail food environments for toddlers are the powerful influence of the FI (including the retailers) and the lack of government support. Many stakeholders expressed the opinion that children’s health is not being prioritized due to the government favoring business (i.e., food-industry profits), over public health. Adequacy of current policy, discussion of options suggested by stakeholders and how they fit with current evidence, as well as barriers to progress, as mentioned by stakeholders, will be discussed.

### Adequacy of policy and regulation of toddler retail food environments

Global research demonstrates that due to a lack of policy action, retail food environments for young children are not health promoting [28, 29]. This sentiment was supported by the Australian stakeholders interviewed for the current study. Meaningful progress toward healthier retail food environments for toddlers is important as food consumption patterns from early childhood can track into later childhood and even adulthood [4,30]. Limiting exposure to non-health promoting retail food environments in the early formative years is therefore crucial.

### Stakeholder suggested policy options to create healthier retail food environments for toddlers

The 2 main options (regulation and nutrition literacy education) suggested by stakeholders as possible solutions may be reflecting what stakeholders perceive as the actual solution (regulation), vs. what they perceive as achievable (education). Despite some coherence among stakeholders around the use of nutrition-content claims and compositional limits, the divergence of support between stakeholder groups for mandatory vs. voluntary regulation was clear. This difference of opinion between FI and public health stakeholders is well established [31]. Public health advocates support mandatory regulation as it ensures public health is the driving factor, as compared to voluntary regulation, which typically fails to produce a public health benefit [32, 33]. Unfortunately, lack of unity is why many public health focused policy options fail [34], as a cohesive voice is needed to garner political will, in addition to removing industry from public health decision making.

Many governments prefer to fund education-based initiatives, as they shift responsibility from the government and impose personal responsibility for change [26], often through dietary guidelines and food labels [35]. However, evidence demonstrates that, unless combined as part of a multifaceted approach, educational campaigns have limited efficacy to improve population health, and can lead to population inequities [26, 36]. It was therefore interesting that nearly all stakeholders in this study recommended nutrition education as an option to improve the healthiness of retail food environments for toddlers. Stakeholders may have mentioned education due to education being a default public health approach [35]. In general, nutrition-based education strategies can be viewed as an important component of a broad, multi-component government policy action, but are not sufficient on their own.

Ongoing monitoring of population food intakes and compliance with existing food regulations are important public health tools. While there is a current update of the Australian Dietary Guidelines in progress, this is not likely to be completed until 2025 [37], leaving the current guidelines potentially out of date. In addition, there is limited dietary data available on toddler (inclusive of 1 to 3 y) dietary intakes in Australia [38], with the most recent population level data from 2017 and including toddlers aged 2–3 y only [39]. Stakeholder discussion around more rigorous enforcement of breaches of the food standards code emphasizes the inadequacy of the current system to monitor and enforce food system breaches. Stakeholders mentioned that long-term monitoring and compliance are often not factored into the public health workforce, or are inadequate, which is a well evidenced argument [20]. To ensure that policies are based on sound evidence, it is crucial to incorporate more robust observational, interventional and surveillance data into the standards for policy change. Additionally, governments must have the necessary technical expertise, resources, and authority to effectively implement and enforce these policies.

### Potential barriers

Highlighting the major role the FI plays in creating retail food environments for toddlers, was the overwhelming agreement among stakeholders that the FI was a barrier to progress. This demonstrates the perception that corporate power is well integrated in Australian food policy [40]. Without comprehensive public health focused, government led policies, the FI can conduct themselves autonomously, within the constraints of the current, outdated regulations. There was polarity between FI and other stakeholder discussions around the barrier of the FI, which aligns with previous food policy research involving diverse stakeholder groups [41]. The FI can, and should become a facilitator to health, and use their skills and power to produce and promote healthy foods.

Stakeholders in this study also perceived the government as a main actor with the power to enact, as well as impede progress on policy action. Evidence supports this sentiment [42]. Lack of regulation in Australia has resulted in retail shelves filled with sweet, UP foods [7], as well as squeeze pouches [43] for toddlers. Infants have an innate preference for sweet, smooth textured foods [44], but the continued consumption of these into the toddler years may result in feeding difficulties and nutrient deficiencies due to lack of the development of chewing ability and reduced dietary variety [45,46]. Packaged foods for toddlers are marketed for their convenience benefits [7,47] but should also highlight the potential risks and negative outcomes from continued use. Currently in Australia, regulations allow toddler milks to proliferate. While toddler milks are not intended for a healthy population [48,49], they are marketed as being essential for healthy, growing toddlers [9,50], and thereby deceive consumers. Strong, government policy action is essential to create healthy, sustainable and equitable retail food environments for young children [35].

Research has shown that most national surveillance systems to monitor food systems are outdated or under resourced [35]. The time taken to develop any new standard or introduce a change to the food standards code is a major barrier for progress, as illustrated by the infant formula standard review (2.9.1), which has been ongoing since 2012 [16]. There is a mismatch between what foods are available and the standards that are regulating them, which is exacerbated by significant delays to regulatory updates, as innovation progresses, with non-existent or outdated food standards relevant to toddler foods and milks. In addition, FSANZ commenced a review of added sugars labeling in June 2021, with no agreed outcome to date [51]. The limited response from government stakeholders to participate in this study may be indicative of the policy priority afforded this issue at present. It is worth noting that this study coincided with a change in federal government, but no state government representation could be achieved either.

### Strengths and limitations

This was the first study to provide insight into stakeholder perceptions regarding the healthiness of retail food environments for toddlers (that we are aware of). The current study had a broad representation of stakeholders, many of whom have considerable experience in their field.

There was a high response and participation rate from those stakeholders who were contacted from FI which is a strength of this study. However, there was a low participation from government as well as NGO’s, which is a limitation of the study in that these stakeholders would most likely have considerable insight into policy feasibility as well as the political appetite for policy change in Australia. All interviews were conducted with stakeholders who had a nutrition or public health background or similar training (including all FI stakeholders), which also may be a limitation, as stakeholders from other departments such as marketing may have held different viewpoints. Stakeholders who did not participate in this research may have differing views from those who did.

## Conclusion

According to stakeholders, advocacy efforts toward healthy retail food environments for toddlers in Australia are being undermined due to government inaction and food-industry interference. In order to create equitable and sustainable healthy retail food environments for Australian toddlers, a multifaceted approach including mandatory regulation and nutrition education should be considered.

## Conflict of interest

None.

## Funding

This research received no specific grant from any funding agency, commercial or not-for-profit sectors.

## Ethical standards disclosure

This study was conducted according to the guidelines laid down in the Declaration of Helsinki and all procedures involving research study participants were approved by the Deakin University Human Ethics Advisory Group: HEAG-H 164_2021. **Verbal** informed consent was obtained from all participants, with consent witnessed and formally recorded.

## Data availability

Data described in the manuscript will be made available upon request.
