# Supplementary material for: Australian Stakeholder Perspectives on Healthier Retail Food Environments for Toddlers—The Era of “Band Aids and Small Inroads”
Source: Curr Dev Nutr. 2023 Dec 9;8(1):102060. doi: 10.1016/j.cdnut.2023.102060 (PMC10806265; doi:10.1016/j.cdnut.2023.102060)
Supplement: Multimedia component 1 [file mmc1.pdf]

Interview protocol for study: Policy options and barriers to healthier retail food environments for toddlers: An Australian perspective, HEAG-H 164_2021

Participant name:

Participant number:

Date:

Introduction

This interview should last for no longer than 45 minutes. You may have read the **Plain Language Statement** already, but if you haven't, please let me know and I can go over it with you. Also, just to reiterate that this will be confidential, with your name or organisation not being disclosed or published.

The aim of this research is to discuss options (policy and non-policy related) and barriers to creating healthier retail food environments for young children in Australia, more specifically toddlers aged 1-3 years, in the context of toddler foods and milks. As someone who has knowledge or experience in this area, I would like to ask you some questions relating to this.

Do you have any questions before we begin?

Do you agree to participate in this study?

Do you agree to the interview being recorded?

Thank you for agreeing to participate. I will start the recording now.

<u>Background questions:</u>

FYI – To clarify:

Retail food environments are supermarkets, chemists, or stores where toddler foods and milks are sold.

By toddler foods I mean any packaged foods specifically labelled for children aged 1-3 years, which are often found next to infant foods in the baby aisle of supermarkets. By toddler milk I mean toddler formulas or the powdered tins of milk found next to infant formulas often labelled as stage 3 or 4 or toddler milk, again found in the baby aisle of supermarkets.

Firstly, I would like to understand a little more about your current and previous research or experience in this area.

1. What is your current role? How long have you been in your current role relevant to this research?

OR

2. Have you been in previous roles that were relevant to this research? If yes, how long ago and how long were you in that area for?

3. Can you tell me about the kinds of activities **you are** involved in relating to toddler foods and/or milks? (can be outside your organisation)

4. What activities does **your organisation** participate in that relates to toddler foods and/or milks?

- You mentioned x, can you please elaborate on what that involves?
- *Further questions in relation to other activities mentioned*

Now I would like to ask you some questions about the current toddler food and milk policy and regulation environment in Australia….

<u>Topic questions</u>

<u>Current policy/regulatory environment of toddler foods and/or milks</u>

5. What is your perception or awareness of current food policy or regulations for toddler foods and milks in Australia?

If you are not fully aware of the regulations that is ok. If you would like me to provide you with an overview I can do that if that would be helpful.

6. Do you feel that current status of policy and regulation of toddler foods and milks adequately promote healthy retail food environments for young children in Australia?

- Why/why not? Please elaborate/explain

7. Are you aware of any policy or regulatory actions in other countries that you feel adequately promote healthy retail food environments for young children? If yes, please elaborate.

- Have you been involved in any of these?

Now I would like to ask you some questions about options and barriers to policy and regulatory changes…

Policy Options/barriers

8. In your opinion, and based on your expertise or interest and involvement in retail food environments for young children, specifically toddler foods and milks, what do you think are some options (policy or non-policy related) to promote healthier retail food environments for young children?

- Prompt if not brought up – what are your thoughts on not permitting claims on toddler foods and milks? Not permitting cross promotion of infant formulas on toddler milks? (if relevant) A balance of positive and negative attribute claims? Align with infant foods and formulas?
- Prompt if not brought up – what are your thoughts on the added sugar labelling discussion that is occurring? Do you think the outcome will have an impact on this area?
- **If a prompt is needed**… other participants have suggested x, or xx has been tried/considered in other countries, what are your thoughts? Do you think this would be feasible?

9. Do you think there would be any barriers to the options you have mentioned?

- Do you think there are any stakeholders or groups that may oppose the options you have suggested?
- Do you think there will be any stakeholders or groups that will be negatively affected if these changes were implemented in Australia?
- Do you think there are ways around these barriers?

10. What do you perceive as the main benefits of your proposed options?

Other questions

11. Are there any people or specific organisations or groups that stand out to you as greatly influencing or driving policy around this issue?

General questions:

12. Is there anything else of importance that we haven't discussed today?

13. Are there any documents that you would be interested in sharing or referring me to in relation to this topic?

14. Is there anyone else who you consider to be 'knowledgeable and/or influential' on the subject that we should interview?

15. If I have follow-up questions, are you ok if I come back to you?

Is there anything else you would like to mention?

Just to let you know the next steps. I will finish up the interviews, and once transcribing and analysis is complete I will plan to submit to a peer reviewed journal. So you can expect to hear from me with some results in around 6 months.

Thank you kindly for your time
